# Aberrant Upregulation of Indoleamine 2,3-Dioxygenase 1 Promotes Proliferation and Metastasis of Hepatocellular Carcinoma Cells via Coordinated Activation of AhR and β-Catenin Signaling

**DOI:** 10.3390/ijms222111661

**Published:** 2021-10-28

**Authors:** Chih-Ta Chen, Pei-Hua Wu, Chia-Chi Hu, Hsiao-Ching Nien, Jin-Town Wang, Jin-Chuan Sheu, Lu-Ping Chow

**Affiliations:** 1 Graduate Institute of Biochemistry and Molecular Biology, College of Medicine, National Taiwan University, No. 1, Jen-Ai Rd, Taipei 100, Taiwan; d02442004@ntu.edu.tw (C.-T.C.); peihua8455@gmail.com (P.-H.W.); danielhu8284@gmail.com (C.-C.H.); 2 Department of Family Medicine, National Taiwan University Hospital, Taipei 100, Taiwan; celian0916@gmail.com; 3 Liver Disease Prevention and Treatment Research Foundation, Taipei 100, Taiwan; jcsheu@ntu.edu.tw; 4 Department of Microbiology, College of Medicine, National Taiwan University, Taipei 100, Taiwan; wangjt@ntu.edu.tw; 5 Department of Internal Medicine, National Taiwan University Hospital, Taipei 100, Taiwan

**Keywords:** hepatocellular carcinoma, proliferation, metastasis, IDO1, kynurenine, AhR, β-catenin, Src, PTEN, Akt

## Abstract

Hepatocellular carcinoma (HCC) is the fourth most common cause of cancer-related death worldwide. Chronic liver inflammation due to hepatitis virus infection and other major effectors is a major risk factor of HCC. Indoleamine 2,3-dioxygenase 1 (IDO1), a heme enzyme highly expressed upon stimulation with proinflammatory cytokines such as interferon-γ (IFN-γ), is activated to modulate the tumor microenvironment and potentially crucial in the development of certain cancer types. Earlier studies have majorly reported an immunomodulatory function of IDO1. However, the specific role of IDO1 in cancer cells, particularly HCC, remains to be clarified. Analysis of The Cancer Genome Atlas Liver Hepatocellular Carcinoma (TCGA LIHC) dataset in the current study revealed a significant correlation between IDO1 expression and HCC. We further established inducible IDO1-expressing cell models by coupling lentivirus-mediated knockdown and IFN-γ induction of IDO1 in normal and HCC cells. In functional assays, proliferation and motility-related functions of HCC cells were compromised upon suppression of IDO1, which may partially be rescued by its enzymatic product, kynurenine (KYN), while normal hepatocytes were not affected. Aryl hydrocarbon receptor (AhR), a reported endogenous KYN receptor, is suggested to participate in tumorigenesis. In mechanistic studies, IDO1 activation promoted both AhR and β-catenin activity and nuclear translocation. Immunofluorescence staining and co-immunoprecipitation assays further disclosed interactions between AhR and β-catenin. In addition, we identified a Src-PTEN-PI3K/Akt-GSK-3β axis involved in β-catenin stabilization and activation following IDO1-mediated AhR activation. IDO1-induced AhR and β-catenin modulated the expression of proliferation- and EMT-related genes to facilitate growth and metastasis of HCC cells. Our collective findings provide a mechanistic basis for the design of more efficacious IDO1-targeted therapy for HCC.

## 1. Introduction

Hepatocellular carcinoma (HCC) accounts for >80% of primary liver cancers worldwide [1]. Liver cancer is the fourth most common cause of cancer-related death globally and ranks sixth in terms of incidence [2]. Based on projections by the World Health Organization, more than one-million deaths from liver cancer are estimated by 2030 [3]. With a 5-year survival rate of ~18%, liver cancer is the second most lethal tumor after pancreatic cancer in the US [4]. In Taiwan, liver cancer ranks fourth in incidence and second as a cause of cancer-related mortality. In view of the high mortality and incidence rates, comprehensive analysis of the mechanisms underlying pathogenesis and development of HCC is critical to improve patient survival.

The major known risk factors contributing to HCC include chronic hepatitis B virus (HBV) and hepatitis C virus (HCV) infection, alcoholic cirrhosis, exposure to aflatoxin B1, non-alcoholic fatty liver disease (NAFLD), or any form of cirrhosis leading to chronic liver damage [5,6,7,8]. Subsequent damage-induced chronic inflammation and further cirrhosis ultimately contribute to liver carcinogenesis. Thus, regulation of the immune system and host cell responses play critical roles in the pathogenesis of HCC [9]. For instance, tumor immune escape and immune tolerance resulting from an immunosuppressive tumor microenvironment serve as key factors in the development and progression of HCC [10,11]. Tumor cells may present immunosuppressive surface ligands or release specific cytokines to suppress the host immune response.

Recent studies indicate that indoleamine 2,3-dioxygenase 1 (IDO1) is significantly elevated under liver inflammation conditions and potentially involved in the regulation of immunity [12,13]. IDO1 is reported to modulate the tumor microenvironment, induce immune tolerance to tumors, promote immune escape, and contribute to HCC progression [14,15]. Overexpression of IDO1 in various cancer types (including liver, colorectal, breast, and ovarian cancer) has been demonstrated [16]. IDO1 has been characterized as a heme-containing enzyme in humans that acts as a rate-limiting factor in the metabolism of L-tryptophan to kynurenine (KYN) [17]. The utility of KYN and IDO1 levels as useful prognostic markers for liver and colorectal tumors and melanoma is extensively documented [16,18].

Other than its role in modulation of the immune microenvironment, recent findings indicate that IDO1 promotes cancer cell development through effects on proliferation and metastasis [19,20]. Earlier preclinical studies have reported that single-dose or combined treatment with the IDO1 inhibitor, 1-methyl-tryptophan, induces a significant reduction in tumor cell proliferation [21,22]. IDO1 was shown to be overexpressed in 35.5% resection samples from HCC patients with significant tumor metastasis and poor prognosis [23]. The collective studies to date implicate correlation of overexpression and increased enzymatic activity of IDO1 with the development and progression of HCC. However, the specific roles and downstream mechanisms of IDO1 and its metabolite, KYN, in HCC remain to be established.

In this study, we investigated the involvement of IDO1 and its downstream pathway in HCC by implementing interferon-γ (IFN-γ)-modulated IDO1-inducible models in control and IDO1 knockdown HCC and normal hepatic cells as a comparison. Experiments using this cellular model platform showed that suppression of IDO1 activity inhibits proliferation, migration and invasion of HCC cells but not normal hepatocytes. Elevated activity of IDO1 and its metabolites activated the downstream mediators, aryl hydrocarbon receptor (AhR) and β-catenin. In addition, activation of β-catenin could be induced via stimulation of the PI3K/AKT pathway by AhR-mediated Src activation and PTEN inhibition. Finally, activation of AhR and β-catenin modulated the expression of downstream cell proliferation- and EMT-related molecules to promote proliferation and metastasis of IDO1-overexpressing HCC cells. Our collective findings highlight the significance of IDO1 in HCC pathogenesis from a tumor cell perspective and support the utility of IDO1-targeted treatments as an effective therapeutic option for HCC.

## 2. Results

### 2.1. IDO1 Expression Is Highly Correlated with HCC Progression

**Figure 1:**
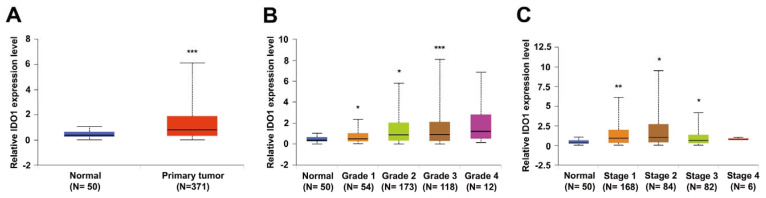
IDO1 expression is significantly elevated in HCC patients. Relative expression levels of IDO1 were compared between HCC tumor and normal tissues based on analysis of a liver hepatocellular carcinoma (LIHC) cohort obtained from the TCGA database via UALCAN platform. Groups were further stratified according to (**A**) sample type, (**B**) individual tumor grade, and (**C**) individual cancer stage (*, *p* < 0.05; **, *p* < 0.01; ***, *p* < 0.001).

To validate the clinical relevance of IDO1 in pathogenesis of HCC, we analyzed data from the liver hepatocellular carcinoma (LIHC) cohort from The Cancer Genome Atlas (TCGA) database retrieved and processed via the UALCAN portal [24]. Comparison of transcriptional expression of IDO1 between tumor and normal tissues revealed significant elevation of IDO1 (*p* < 0.001) in tumor samples (Figure 1A). Further analysis based on tumor grade classification showed grade-dependent upregulation of IDO1 in tumors (grade 1 to 3) compared to control counterparts (Figure 1B). Additionally, in analysis of IDO1 expression based on cancer stage, we observed an increase in IDO1 at stages 1 to 3, with stage-dependent elevation mainly in the early to early-intermediate stages (stage 1 to 2; Figure 1C). These results clearly indicate that IDO1 contributes to HCC progression, supporting further exploration of its functional significance in tumorigenesis.

### 2.2. IDO1 Activity Is Implicated in HCC Proliferation and Metastasis

**Figure 2:**
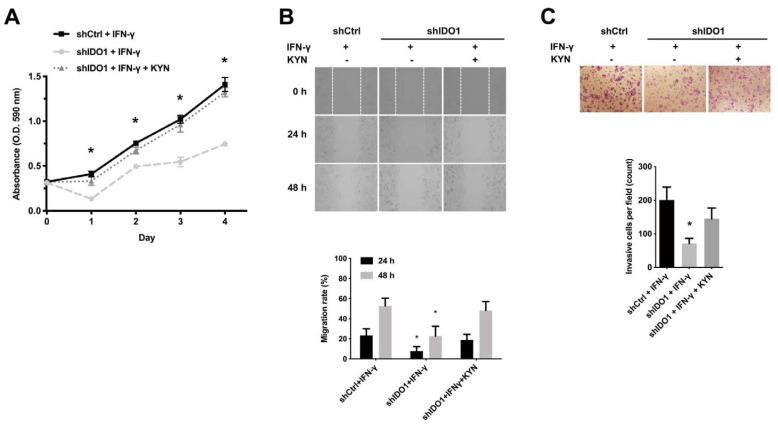
Upregulation of IDO1 activity promotes proliferation, migration and invasion of HCC cells. (**A**) Promotion of HCC cell proliferation is IDO1 activity-dependent. Knockdown of IDO1 significantly suppressed the proliferation of shIDO1 cells compared to shCtrl cells, while KYN recued the growth of shIDO1 counterparts. Viability of IDO1 knockdown and control HuH-7 cells under IFN-γ (40 ng/mL) treatment with or without KYN (100 µM) rescue monitored for four days via MTT assay. Plots depict cumulative absorbance versus number of days. (**B**) Knockdown of IDO1 significantly inhibited the migration of shIDO1 cells compared to the shCtrl cells. The lost migratory ability was rescued by KYN. Wound healing assay of shCtrl and shIDO1 HuH-7 cells treated with IFN-γ (40 ng/mL) with or without KYN (100 µM) rescue supplemented with 1% FBS. Upper, representative micrographs showing cells migrating into the gap at 0 h, 24 h, and 48 h after removal of the culture insert at 200× magnification. Lower, quantification of migration rates from three independent replicates. (**C**) Knockdown of IDO1 significantly inhibited the invasion of shIDO1 cells compared to the shCtrl cells. Induction by KYN restored the invasiveness of shIDO1 cells. Transwell invasion assay of control and IDO1 knockdown HuH-7 cells under IFN-γ (40 ng/mL) treatment with or without KYN (100 µM) rescue. Upper, cells in the central field of each insert visualized via light microscopy at 200× magnification after 72 h. Lower, invading cells quantified via manual counting. Data are presented as mean ± SD of three independent experiments (shCtrl, control shRNA; shIDO1, shRNA against IDO1; *, *p* < 0.05).

To clarify the specific functional role of IDO1 in HCC, IFN-γ-dependent induction of IDO1 expression and shRNA-mediated IDO1 knockdown in a HuH-7 cellular model were conducted as shown in Figure S1A. The activity of induced IDO1 was examined by measuring the released enzymatic product, kynurenine, in the condition medium (Figure S1B). Expression and activity of IDO1 in control cells were IFN-γ-inducible, while expression in the knockdown cell counterparts was nearly abolished. Following validation of IFN-γ-inducible IDO1 activity, cell proliferation and motility assays were performed. The growth of shIDO1 HuH-7 cells was significantly suppressed relative to shCtrl HuH-7 cells under IFN-γ stimulation (Figure 2A). Notably, shIDO1 HuH-7 cell growth was restored to nearly control cell levels following rescue with the enzymatic product KYN (100 μM). The migration and invasive abilities of shIDO1 HuH-7 cells with suppression of IDO1 expression and activity were significantly inhibited (Figure 2B,C). However, suppression of migration and invasiveness of IDO1 knockdown cells was greatly compensated by the activity-derived product KYN. To further examine the functional role of IDO1 in other HCC cell lines and in normal liver cell as comparison, we collected three other HCC cell lines, Hep3B, HepG2, Sk-Hep-1, and Ph5Ch8 normal hepatocyte cells. The expression levels of IDO1 under IFN-γ induction were determined by immunoblotting (Figure S2A) and Ph5Ch8 and Sk-Hep-1 were selected for further examination. As a comparison, functional assays related to cell growth and cell motility were performed to validate the importance of IDO1 in HCC cells. As shown in Figure S2B**,** suppression of IDO1 expression prominently inhibited the proliferation of Sk-Hep1 cells. There is a difference being aware that KYN marginally rescued the shIDO1 Sk-Hep1 cells. Though differences are small, the result is statistically significant. On the contrary, knockdown of IDO1 seemed not to affect the growth of Ph5Ch8 normal hepatic cells (Figure S2C). Correspondingly, inhibiting the function of IDO1 impaired the migration and invasiveness of Sk-Hep1 cells while Ph5Ch8 cell function was not changed (Figure S2D,E) Similarly, the efficacy of KYN rescue on invasiveness of Sk-Hep1 cell was limited. Nevertheless, our results indicate that IDO1 promotes proliferation, migration, and invasion of HCC cells, which are mainly enzymatic activity-dependent.

### 2.3. AhR and β-Catenin Are Potential Downstream Mediators of IDO1 Tumor-Promoting Activity

**Figure 3:**
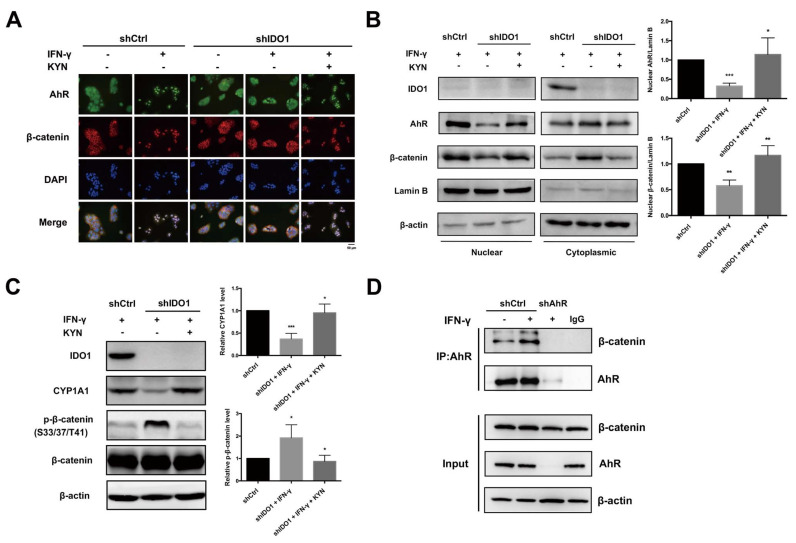
IDO1 activation induces downstream AhR and β-catenin nuclear translocation, activity and mutual interactions. (**A**) Induction of IDO1 simultaneously promoted nuclear translocation of AhR and β-catenin in shCtrl HuH-7 cells. Knockdown of IDO1 impaired nuclear translocation of both molecules in IDO1 knockdown cells, which was rescued by KYN treatment. Control and IDO1 knockdown HuH-7 cells were treated with or without IFN-γ (40 ng/mL) for 48 h in the presence or absence of KYN (100 µM), and AhR and β-catenin localization were examined via IF staining. Cell nuclei were stained with DAPI (blue), AhR with FITC-labeled antibody (green), and β-catenin with TRITC-labeled antibody (red). Scale bar, 50 μm (**B**) Nuclear AhR and β-catenin were significantly increased in shCtrl cells under IDO1 activation compared to shIDO1 counterparts. Nuclear translocation of AhR and β-catenin in control and IDO1 knockdown HuH-7 cells with or without KYN (100 µM) rescue under IFN-γ (40 ng/mL) stimulation for 48 h examined via the cell fractionation assay. Left, fractionated samples were separated via SDS-PAGE and analyzed via immunoblot with the specified antibodies. Right, relative changes in nuclear AhR and β-catenin quantified using Lamin B as a normalization control. (**C**) Induction of IDO1 significantly increased the expression of CYP1A1 and decreased the phosphorylation of β-catenin in shCtrl cells relative to shIDO1 counterparts. Examination of AhR and β-catenin activity under IFN-γ (40 ng/mL) induction in control, shIDO1 or KYN (100 µM) rescued HuH-7 cell groups by comparing downstream CYP1A1 and p-β-catenin (S33/37/T41) expression via immunoblotting, left. Right, relative changes in CYP1A1 and p-β-catenin quantified using β-actin or β-catenin as normalization control, respectively. (**D**) Activation of IDO1 significantly increased the co-immunoprecipitated β-catenin in shCtrl HuH-7 cells. Interactions of AhR and β-catenin under IDO1 activation via IFN-γ (40 ng/mL) induction, validated using co-immunoprecipitation assay and subsequent immunoblot analyses. All statistical data were calculated from three independent replicates (shCtrl, control shRNA; shIDO1, shRNA against IDO1; shAhR, shRNA against AhR; *, *p* < 0.05; **, *p* < 0.01; ***, *p* < 0.001).

Our data suggest that KYN produced by IDO1 serves as an important intermediate in HCC cell function. In addition, KYN is an endogenous ligand for the intracellular receptor, AhR, and shown to directly activate and promote translocation of AhR to the nucleus for gene regulation [20]. Accordingly, we investigated the influence of IDO1 activity on AhR localization. Following induction of IDO1 expression with IFN-γ, we observed a shift in AhR from the cytoplasm to nucleus in shCtrl HuH-7 cells, while localization of AhR remained restricted to the cytoplasm in IDO1-inhibited cells (Figure S3A). Notably, however, translocation of AhR from the cytosol to nucleus was reactivated when loss of IDO1 activity was compensated by KYN. KYN alone could also promote nuclear translocation of AhR in shCtrl HuH-7 cells without IFN-γ stimulation in a dose-dependent manner (Figure S3B). Previous experiments suggest that IDO1 activation stimulates β-catenin activity and nuclear localization, along with transcription of its target genes [25]. As shown in Figure 3A, β-catenin co-translocated to the nucleus with AhR in control HuH-7 cells following IDO1 activation by IFN-γ, which was not observed in IDO1-depleted cells. KYN rescue in IDO1 knockdown cells and KYN alone similarly induced simultaneous nuclear translocation of β-catenin and AhR (Figure 3A and Figure S3C). In parallel, we also examined the translocation of AhR and β-catenin under modulation of IDO1 activity in Sk-Hep1 cells. Compared to the control cells, induction of IDO1 partially increased the nuclear translocation of AhR and β-catenin in Sk-Hep1 cells relative to HuH-7 counterparts (Figure S4A). Suppression of IDO1 expression impeded the translocation of both molecules while the translocation was partially rescued by KYN. Analogous to the above results, nuclear translocation of AhR and β-catenin under IFN-γ stimulation conditions was inhibited in IDO1 knockdown HuH-7 cells relative to control cells, which could be rescued with the addition of KYN. Lamin B and β-actin were employed as controls for purity of nuclear and cytosolic fractions, respectively (Figure 3B and Figure S3D). To validate activation of AhR and β-catenin following nuclear translocation, expression of the downstream target of AhR, CYP1A1, and variations in inhibitory phosphorylation of β-catenin at S33/37/T41 were examined. Our results showed suppression of CYP1A1 and increased inhibitory phosphorylation at S33/37/T41 of β-catenin following impairment of IDO1 expression and activity in both HuH-7 and Sk-Hep1 cells (Figure 3C and Figure S4B). AhR and β-catenin activities were restored following KYN rescue in HuH-7 cells but partially in Sk-Hep1 cells (Figure 3C, Figures S3E and S4B). Potential mutual interactions between AhR and β-catenin have been previously reported [26]. In view of the above co-localization results, we examined the possibility of physical interactions between these factors under IDO1 activation. In co-immunoprecipitation experiments, increased interactive binding of AhR and β-catenin was observed under IFN-γ or KYN stimulation, which was lost upon AhR depletion (Figure 3D and Figure S3F).

### 2.4. IDO1 Activates β-Catenin through the Src-PTEN-PI3K/Akt-GSK-3β Axis following AhR Activation

**Figure 4:**
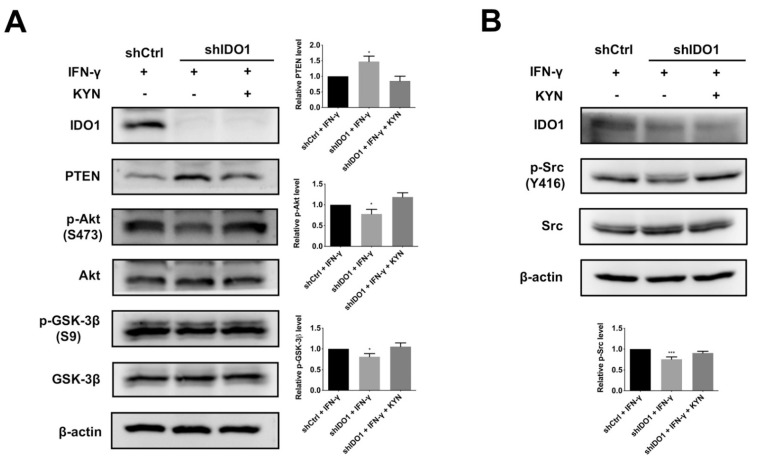
AhR-mediated Src-PTEN-Akt-GSK-3β pathway activation promotes β-catenin activity under IDO1 upregulation. (**A**) Sequential induction of IDO1 and AhR activity inhibited GSK-3β via PTEN suppression and Akt activation. The expression of PTEN was downregulated while the phosphorylation of Akt (S473) and GSK-3β (S9) was upregulated by IFN-γ induction in shCtrl cells or by KYN rescue in shIDO1 cells. Left, shCtrl and shIDO1 HuH-7 cells were induced by IFN-γ (40 ng/mL) with or without KYN (100 µM) rescue. Cell lysates were separated via SDS-PAGE and analyzed via immunoblotting with the specified antibodies. Right, quantitative analysis of alterations in expression levels of PTEN, p-Akt (S473) and p-GSK-3β (S9). (**B**) Promotion of Src activity under IDO1 upregulation and subsequent AhR activation. The phosphorylation of Src (Y416) was significantly increased in shCtrl cells by IFN-γ induction compared to shIDO1 cells. KYN rescue of shIDO1 cells restored the upregulation of p-Src (Y416). Upper, control and IDO1 knockdown HuH-7 cells were stimulated by IFN-γ (40 ng/mL) with or without KYN (100 µM) rescue. Cell lysates were separated via SDS-PAGE and subjected to immunoblot analysis. Lower, quantification of changes in phosphorylation of Src. Band intensities were quantified via densitometry and normalized to β-actin from three independent replicates (shCtrl, control shRNA; shIDO1, shRNA against IDO1; *, *p* < 0.05; ***, *p* < 0.001).

To explore the link between β-catenin and IDO1 activity, a mechanistic study was performed. AhR-induced β-catenin activity through PTEN inhibition and Akt activation has been previously reported [27], along with subsequent inhibition of GSK-3β activity [28]. Accordingly, we postulate that AhR activation by IDO1 triggers inhibition of PTEN, stimulation of Akt, and subsequent suppression of GSK-3β to promote β-catenin activity. As shown in Figure 4A and Figure S6A, IDO1 induction by IFN-γ led to inhibition of PTEN, upregulation of Akt, and promotion of inhibitory Serine 9 (S9) phosphorylation of GSK-3β in both control HuH-7 and SK-Hep1 cells, while this pathway is not altered in Ph5Ch8 normal hepatic cells (Figure S6B). KYN rescue of IDO1 knockdown cells restored the signaling axis to near the level of controls in both HCC cells (Figure 4A and Figure S6A). In keeping with the above findings, knockdown of AhR blocked PTEN-Akt-GSK-3β signaling in HuH-7 cells (Figure S5B). A previous study has reported that Src inhibits PTEN and promotes PI3K/Akt signaling through altering PTEN protein stability [29]. Results from an earlier colon cancer study also suggest that the original AhR-bound Src is released and activated after AhR activation [30]. Accordingly, we examined the hypothesis that AhR activation by IDO1 could stimulate Src and thus inhibit PTEN to promote downstream PI3K/Akt activity. As evident from induction-and-rescue assay, active IDO1 and AhR promoted activating phosphorylation at Tyr 416 (Y416) of Src in both HCC cells but not in the Ph5Ch8 normal hepatic cells (Figure 4B and Figure S6A,B). Additionally, in KYN time-dependent assay, decreased expression of PTEN and elevated Akt, GSK-3β and Src phosphorylation under AhR activation was observed in HuH-7 model (Figure S5A,C). In summary, activation of IDO1 and subsequent AhR release stimulate Src activity, which, in turn, inhibits PTEN and promotes downstream PI3K/Akt-GSK3β signaling, leading to β-catenin activation.

### 2.5. IDO1 Promotes Proliferation and Metastasis of HCC Cells through Modulating Proliferation- and EMT-Related Molecules

**Figure 5:**
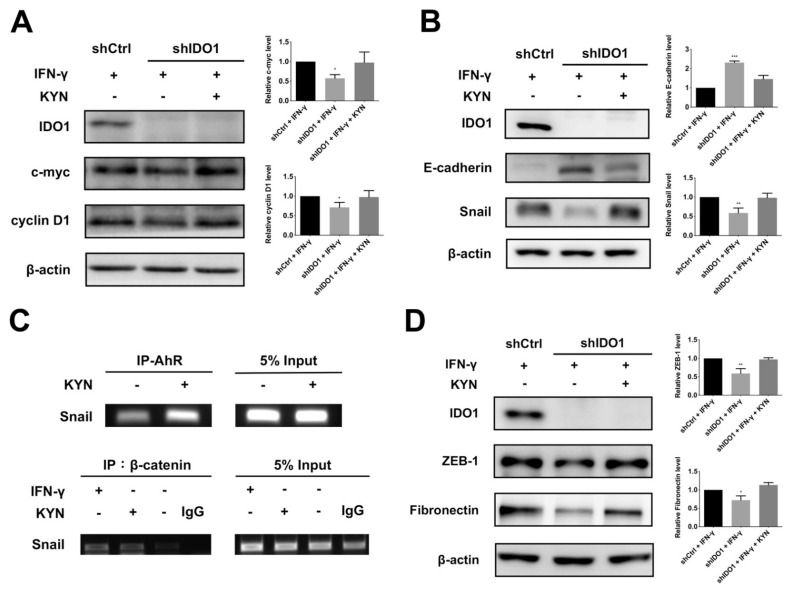
IDO1 enhances HCC proliferation and metastasis through modulation of downstream proliferative and EMT-related markers. (**A**) Expression levels of proliferative markers, c-myc and cyclin D1, under IDO1 and AhR activation. C-myc, cyclinD1 were both upregulated in shCtrl cells by IFN-γ induction relative to shIDO1 cells. Left, control and shIDO1 HuH-7 cells were treated with IFN-γ (40 ng/mL) with or without KYN (100 µM) rescue, and expression of the indicated proteins analyzed via immunoblotting with specific antibodies. Right, relative quantification of immunoblotting results from three independent replicates. (**B**) Evaluation of expression of the EMT regulator, Snail, and epithelial marker, E-cadherin, under IDO1 and AhR activation. Induction of IDO1 increased Snail and decreased E-cadherin expression in shCtrl cells compared to IDO1 knockdown cells. Left, control and shIDO1 HuH-7 cells were treated with IFN-γ (40 ng/mL) with or without KYN (100 µM) rescue. Samples were separated via SDS-PAGE and analyzed via immunoblotting. Right, quantitative analysis of variations in Snail and E-cadherin levels. (**C**) The binding of AhR and β-catenin to the promoter of Snail was significantly increased by IFN-γ or KYN induction in HuH-7 cells. Upper, control HuH-7 cells were examined for AhR binding at the Snail promoter region via ChIP assay following induction with or without KYN (100 µM) for 8 h. Cell lysates were immunoprecipitated with anti-AhR antibody. Specific primers for Snail promoter were used to analyze purified precipitates or input DNA for Snail-specific signals. Lower, analysis of β-catenin binding to Snail promoter region under IDO1 and AhR activation in control HuH-7 cells. Cell lysates were immunoprecipitated with anti-β-catenin antibody. Specific primers for Snail promoter were used for Snail-specific signal. (**D**) ZEB-1 and fibronectin were significantly upregulated under IDO1 activation in shCtrl cells compared to shIDO1 counterparts. Immunoblot analysis of expression levels of two Snail-regulated mesenchymal markers, ZEB-1 and fibronectin, under IDO1 and AhR induction, left. Right, quantification of alterations in expression levels of ZEB-1 and fibronectin. All statistical data were calculated from three independent replicates (shCtrl, control shRNA; shIDO1, shRNA against IDO1; *, *p* < 0.05; **, *p* < 0.01; ***, *p* < 0.001).

While the above results clearly indicate that IDO1 activation promotes proliferation of HCC cells, the downstream mechanisms remain unclear. β-Catenin is reported to regulate the expression of several proliferative genes, such as c-myc and cyclin D1 [31]. Accordingly, we investigated whether IDO1-induced β-catenin facilitates HCC proliferation through upregulation of c-myc and cyclin D1. Induction of IDO1 activity led to upregulation of c-myc and cyclin D1 in control cells relative to shIDO1 HuH-7 cells (Figure 5A). In a time-dependent KYN induction experiment, increase in c-myc and cyclin D1 under conditions of IDO1-AhR activation was validated (Figure S7A). In a parallel examination on Sk-Hep1 cells however, activation of IDO1 only promoted the expression of CyclinD1 (Figure S7D). Based on the finding that the cell cycle-related modulator, cyclin D1, is affected by IDO1 activity, we further focused on the impact of IDO1 on cell cycle checkpoint molecules, such as cyclin D1-CDK4/6 inhibitor, p21^WAF/Cip1^, p53, activator of p21^WAF/Cip1^ and p27^kip1^. In both the knockdown-induction model and time-dependent induction assays, upregulation of IDO1 and AhR led to suppression of these cell cycle checkpoint proteins in HuH-7 cells, while only p27^kip1^ was significantly modulated by IDO1 activity in the Sk-Hep1 counterparts (Figure S8A–C).

**Figure 6:**
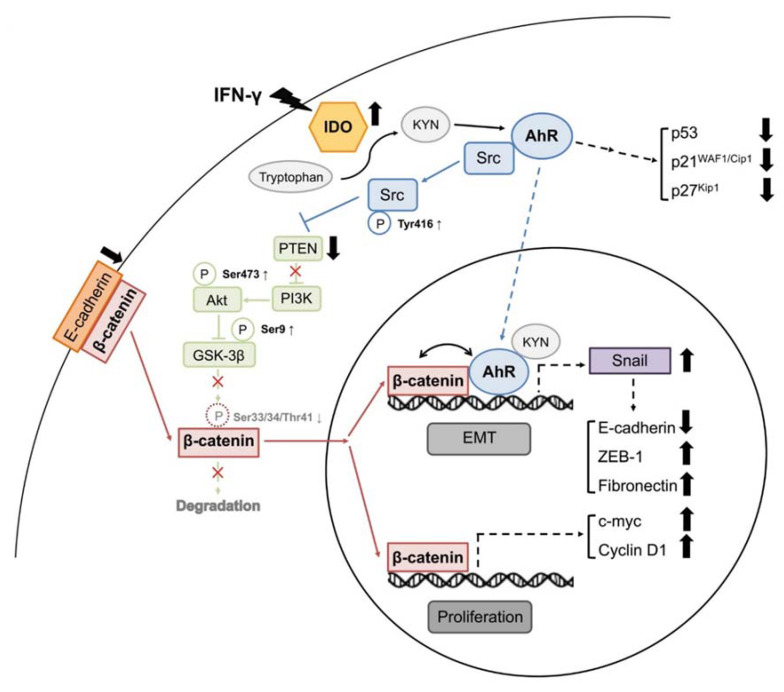
A proposed model of IDO1-induced HCC progression via coordinated promotion of AhR and β-catenin pathways. Activation of IDO1 in HCC promotes activity of the downstream mediator AhR. Activated AhR subsequently upregulates Snail and promotes β-catenin activity through Src-mediated Akt activation and GSK-3β inhibition. Alone or together, IDO1 activity-induced AhR and β-catenin modulate the expression of Snail-regulated EMT molecules, proliferative markers, and cell cycle checkpoint inhibitors, though with cell diversity, to promote proliferation and facilitate metastasis of HCC cells. Dash line stands for versatile modulation.

Suppression of IDO1 activity clearly inhibited metastasis-related functions (migration and invasion) of HCC cells. Accordingly, we further focused on the downstream mechanisms following IDO1-AhR induction. Several studies have highlighted the importance of a cellular process known as epithelial-mesenchymal transition (EMT) in cancer metastasis. Loss of cell adhesion is an important early event of EMT, especially at E-cadherin-mediated adherent junctions. Snail, a well-known EMT-promoting transcription factor, is a potential target gene under AhR regulation [32] shown to suppress E-cadherin expression during tumor metastasis [33]. Accordingly, we examined whether activation of IDO1 and subsequent AhR could augment Snail and attenuate E-cadherin expression to promote EMT and facilitate motility of HCC cells. Upon induction of IDO1 in control HuH-7 cells, E-cadherin expression was inhibited while Snail was upregulated, compared to IDO1 knockdown cells (Figure 5B). Similar results were observed in Sk-Hep1 cells but the effect of KYN rescue is marginal (Figure S7D). Analogous results were obtained in the time-dependent KYN induction experiment in HuH-7 cells, validating the dependency of EMT promotion on AhR activity following IDO1 activation (Figure S7B). On the other hand, potential interactions between AhR and β-catenin in the nucleus were noted. Accordingly, binding of AhR and β-catenin at the promoter region of Snail was further examined via ChIP assay. As shown in Figure 5C, both AhR and β-catenin displayed increased binding to the promoter region of Snail in the presence of KYN or IFN-γ. A number of Snail-regulated EMT-related molecules, such as ZEB-1 and fibronectin, were also upregulated upon IDO1 and AhR activation in control HuH-7 cells, while only fibronectin was regulated by IDO1 activity in Sk-Hep1 counterparts (Figure 5D and Figure S7C,E). Our results support a mechanistic pathway whereby IDO1 upregulation induces activation and nuclear translocation of AhR and β-catenin, which cooperatively modulate downstream proliferation- and EMT-related genes to promote proliferation and metastasis of HCC cells (Figure 6).

## 3. Discussion

HCC, one of the most lethal malignancies worldwide, has been characterized as a chronic liver damage-induced disease resulting from HBV and HCV infection or other non-viral factors [34,35]. Chronic hepatocyte damage can lead to fibrosis and cirrhosis, ultimately contributing to HCC development. IDO1, one of the many liver damage-induced molecules, is reported to play critical roles in immune homeostasis and immune tolerance [36,37]. IDO1 is involved in several pathological conditions, including atherosclerosis, autoimmunity, infections, and cancer [36]. Several studies have confirmed IDO1 expression in various cancer types, including lung, colorectal, prostate, breast, and ovarian cancers, in association with poor patient prognosis [16]. IDO1 has been frequently detected in tumor specimens of patients with HCC (97.3%) [38]. However, the correlation of IDO1 expression with HCC prognosis and its specific role in tumor development remain controversial [37]. IDO1 is suggested by some research groups to serve as a favorable prognostic marker with a role in anti-tumor immunity, while other studies suggest that IDO1 is implicated in tumor promotion via immunosuppressive effects [38,39]. In this study, we have addressed the functional roles and potential downstream mechanisms of IDO1 in HCC development. 

We initially analyzed data from the HCC cohort of TCGA to ascertain the potential clinical relevance of IDO1 in HCC. One significant finding was marked upregulation of IDO1 mainly from the early to intermediate stages. This early event of anomalous elevation of IDO1 is suggestive of diagnostic potential in HCC. On the other hand, IDO1 expression is positively correlated with tumor grade, suggesting prognostic value in HCC tumor progression. A recent meta-analysis demonstrated high expression of IDO1 in solid tumors indicative of both predictive and prognostic potential [40].

While the diagnostic and prognostic applications of IDO1 in HCC require further clinical validation, its therapeutic value based on immunomodulatory functions has been exploited in many early trials [41]. However, several setbacks and side effects have been reported due to the lack of understanding of the mode of action and complex downstream signaling of IDO1. Here, we have reported previously unspecified effects of IDO1 on HCC and novel findings on its downstream mechanism of action (Figure 6). Functional assays validated the importance of IDO1 in the proliferative and metastatic abilities of HCC cells. Our results are consistent with previous studies showing that IDO1 promotes cancer cell proliferation in animal models and human tumor samples of colon cancer [25,42]. Moreover, knockdown of IDO1 suppressed the metastatic development of cancer cells as demonstrated for KRAS-induced lung carcinoma [43].

We further analyzed downstream molecules potentially involved in tumor-associated functions following IDO1 activation. Activation and potential interactions of β-catenin with AhR were observed. Activation of β-catenin following IDO1 upregulation and subsequent AhR activation were analogous to data obtained with the colon cancer model [25]. In addition, AhR activation by KYN following IDO1 activation led to stimulation of Src, which inhibited PTEN activity, in turn, augmenting PI3K/Akt signaling. Upregulation of Akt induced blockage of GSK-3β, leading to stabilization and activation of β-catenin. AhR activated by bound dioxin-like ligands is reported to free and activate the original bound c-Src [44]. Destabilization and inhibition of PTEN via Src and subsequent activation of the PI3K/Akt cascade have also been indicated in earlier studies [29,45]. In summary, our findings support involvement of the Src-PTEN-PI3K/Akt-GSK-3β axis for β-catenin activation under IDO1 and AhR activation.

Following activation, both AhR and β-catenin translocate to the cell nucleus where they play tumor-promoting roles. Activated nuclear β-catenin is known to enhance the expression of pro-proliferative genes, such as c-myc and cyclin D1 [31]. We further considered the possibility of effects of IDO1-AhR activation on other proliferation-related genes, in particular, those involved in the cell cycle other than cyclin D1. During the cell cycle process, checkpoint monitoring is an important control for proper and accurate cell division and propagation. Among the checkpoint molecules, p53, p21^WAF1/Cip1,^, and p27^Kip1^, which control progression of the cell cycle from G0 through to S phase for maintenance of genetic accuracy and stability, are the most extensively characterized [46,47]. Notably, IDO1-AhR activation significantly suppressed these three checkpoint inhibitors of cell proliferation. AhR activation by TCDD was shown to attenuate p53 function through Mdm2 activation in an earlier study [48]. p21^WAF1/Cip1^ is a downstream target modulated by p53 [46]. UV-induced AhR activation inhibits p27 and compromises early defense systems against skin carcinogenesis [49]. Further studies are required to establish the detailed mechanisms underlying modulation of p53, p21^WAF1/Cip1^, and p27^Kip1^ in HCC cells. In summary, IDO1-AhR-β-catenin activation in HCC cells promotes the expression of pro-proliferative genes with concomitant suppression of cell cycle inhibitory molecules to promote proliferative ability.

A recent study suggests that AhR performs a tumor suppressor-like rather than promoter function in glioblastoma and its suppression facilitates invasion of cancer cells [50]. However, our experiments support a different role of AhR in HCC cells. In addition to promoting proliferation of HCC cells, IDO1 upregulation enhanced the mobility and invasiveness of both HuH-7 and Sk-Hep1 cells. We showed that AhR activated by IDO1 translocated to the nucleus and binds the promoter of Snail in HuH-7 cells, a previously reported downstream target [32]. Upregulated Snail suppresses the expression of E-cadherin, an important effector of EMT during tumor metastasis [33]. E-cadherin expression and stability exert a significant regulatory effect on β-catenin activity [51]. Inhibition of E-cadherin is therefore a critical step in the release and activation of β-catenin. Formation of the E-cadherin/AhR/Skp2 complex and subsequent ubiquitination of E-cadherin induced by kynurenine has been documented in lung cancer and breast cancer cells, indicating a generalized mechanism of E-cadherin suppression by kynurenine in different cancer types [52]. Here, we present a novel mechanistic pathway of β-catenin activation following IDO1/AhR activation via AhR/Snail-mediated E-cadherin suppression in HCC cells. Together, the Src-PTEN-PI3K/Akt-GSK-3β pathway and Snail-mediated E-cadherin suppression form a positive feedback circuit of EMT-promoting β-catenin activation.

On the other hand, we also observed mutual interactions between AhR and β-catenin under IDO1 induction. The molecules bound simultaneously at the promoter of Snail, suggesting cooperative modulation of downstream EMT-related gene expression. Potential interactions and crosstalk between AhR and β-catenin have been highlighted by several investigators [26,53]. In this study, we further examined the effects of IDO1 expression on other Snail-regulated genes. Our results showed upregulation of ZEB-1 and fibronectin, two Snail-modulated mesenchymal markers, during EMT conversion [54]. Potential alterations in other proliferative, EMT and metastatic molecules require further investigation for comprehensive understanding of pathogenic and oncogenic activity of IDO1.

To examine the potential impact of IDO1 expression and activity on HCC and to understand differences in the functional role of IDO1 between HCC and normal liver cells, we performed cell line screening and in advance chose a normal hepatocyte (Ph5Ch8) and an additional HCC cell (Sk-Hep1) for further investigation. From the results of screening, we saw a relative common induction of IDO1 expression among HCC cell lines though with different extents. Similar phenomena were observed in several earlier studies [23,55]. Tumor heterogeneity in cancers has indicated a possible explanation for this variation [56,57,58]. Beyond that, we still validated the functional importance of IDO1 activity induction in HCC from the results of two cell lines. On the other hand, the activity of IDO1 in Ph5Ch8 normal hepatic cells seems not indispensable with little detectable functional relevance. We are also aware of some intriguing diversity on the regulation of downstream molecules after IDO1 and AhR activation among different HCC cells. Although the main Src-PTEN-PI3K/Akt-GSK-3β signaling axis generally remained activated, the activity of AhR alone varied under different cellular contexts. In addition, regulation of downstream targets after AhR and β-catenin activation also showed cell-dependent differences. Incompetence of KYN to restore AhR activity in Sk-Hep1 requires further understanding of the regulation among IDO1-KYN-AhR, but an earlier report has implicated the involvement of other possible regulating mediators of AhR. Functional impairment of the AhR-interacting protein due to genetic mutation disrupt its physical or functional interaction with AhR [59]. Nevertheless, the importance of IDO1 activity and the induced main pathways are still influential in HCC cells apart from minor cell-dependent differences.

In conclusion, we used knockdown-induction HCC (HuH-7 and Sk-Hep1) cell models to ascertain the significance of IDO1 in HCC tumorigenesis and development. The present findings have highlighted a previously unknown role of IDO1 other than immunosuppressive activity in HCC, which may be applicable to other cancer types. We propose a novel mechanism by which IDO1 exerts oncogenic effects in HCC cells that involves two pathways. The first is a proliferative pathway that is majorly activated through AhR-Src-PTEN-PI3K/Akt-GSK-3β-β-catenin signaling and the other is a pro-metastatic pathway that relies on AhR/β-catenin-Snail cooperativity. Together, these two major downstream reciprocal circuits promote the development and progression of HCC through cooperative modulation of proliferation, cell cycle, and EMT genes. Our novel mechanistic findings aid in clarifying the role of IDO1 and provide a molecular basis for the development of highly effective IDO1-targeted therapy for HCC.

## 4. Materials and Methods

### 4.1. Analysis of HCC Clinical Data from The Cancer Genome Atlas (TCGA) Database

To analyze the correlation of IDO1 expression status with HCC development, the UALCAN portal (http://ualcan.path.uab.edu, accessed on 15 July 2019) was adopted directly for the analyses of stratified comparison of IDO1 expression as specified previously [24]. The results were directly extracted and downloaded from the portal. The rationale of analysis design is described briefly as follows. Level 3 RNA-seq data for 31 cancer types, including LIHC, were downloaded. The estimated expression values of genes were transformed into transcripts per million. Patient data were obtained from Genomic Data Commons (https://gdc.cancer.gov/, accessed on 15 July 2019) and established by the portal founder. Expression levels of IDO1 between normal and HCC patients and in different subgroups, including tumor grades and individual cancer stages, were presented as box–whisker plots. The significance of differences was evaluated using t test as stated by the portal developer (designated * *p*< 0.05, ** *p* < 0.01 or *** *p* < 0.001).

### 4.2. Cell Culture, IDO1 Knockdown and Functional Assays

The HuH-7 cell line was obtained from the Health Science Research Resources Bank (JCRB0403, Tokyo, Japan). Normal hepatocyte cells Ph5Ch8 was kindly provided by Professor Helene Min-Yi Liu from Graduate Institute of Biochemistry and Molecular Biology, College of Medicine, National Taiwan University. Hep3B, HepG2 and Sk-Hep-1 were obtained from the American Type Culture Collection. Control, IDO1 knockdown or AhR knockdown HuH-7 and HEK-293T cells were maintained in Dulbecco’s Modified Eagle medium (DMEM; HyClone, Marlborough, MA, USA) supplemented with 10% fetal bovine serum (FBS; HyClone), penicillin (100 U/L), and streptomycin (10 mg/L) in a 37 °C humidified incubator under 5% CO_2_. Ph5Ch8 and other HCC cell lines, including Hep3B, HepG2 and Sk-Hep-1 were maintained in DMEM in the same condition as HuH-7. The target sequences for IDO1 or AhR knockdown are listed in Table S1. Lentiviruses expressing small hairpin RNA (shRNA) against IDO1 (shIDO1), AhR (shAhR), or control shRNA (shCtrl) were produced in HEK293T cells. Medium containing shIDO1, shAhR, or shCtrl viruses was added to HuH-7, Sk-Hep1, and Ph5Ch8 cell cultures. Cell proliferation, nuclear localization, fractionation, wound healing, and invasion assays were performed after confirmation of knockdown efficiency via immunoblotting.

### 4.3. Cell Proliferation, Wound Healing, and Invasion Assays

Cell proliferation and viability were examined using the 3-(4,5-dimethylthiazol-2-yl)-2,5-diphenyltetrazolium bromide (MTT) assay (Sigma-Aldrich; St Louis, MO, USA) and IC_50_ values determined by measuring absorbance at 570 nm. Cell migration ability was assessed with a scratch wound healing assay and the invasive capability of cells evaluated using a Matrigel-coated Boyden chamber assay. The growth, migration and invasion abilities of shCtrl and shIDO1 HuH-7, Sk-Hep1 and Ph5Ch8 cells exposed to IFN-γ (40 ng/mL) with or without KYN (100 μM) rescue were compared as described previously [58].

### 4.4. Immunofluorescence Staining and Immunoblotting

AhR and β-catenin localization changes were examined via immunofluorescence (IF) staining as described in a previous report [58]. Briefly, shCtrl and shIDO1 HuH-7 and Sk-Hep1 cells were treated with or without IFN-γ (40 ng/mL) in the absence or presence of KYN at the indicated doses for 48 h at 37 °C. After treatment, cells were fixed, blocked, and immunostained with antibodies against AhR (Santa Cruz, Dallas, TX, USA) and β-catenin (BD Biosciences, San Jose, CA, USA), followed by fluorescein isothiocyanate (FITC)-labeled (AhR) or tetramethylrhodamine-isothiocyanate (TRITC)-labeled (β-catenin) secondary antibodies. Nuclear staining was conducted with 4′,6-diamidino-2-phenylindole (DAPI) and images captured using an Olympus BX-51 microscope (Olympus, Japan). For immunoblot analysis, HuH-7, Sk-Hep1 and Ph5Ch8 cells were subjected to lentivirus-mediated knockdown of IDO1 or AhR and exposed to IFN-γ (40 ng/mL) or KYN at the indicated doses and times. All samples were lysed in RIPA containing 150 mM NaCl, 5 mM EDTA, 25 mM Tris-HCl, 1% NP 40, 0.1% SDS, 1× protease inhibitor cocktails, pH 8.0. Lysates were separated via SDS-PAGE and transferred to PVDF membranes. Antibodies used for subsequent immunoblotting are listed in Table S2.

### 4.5. Cell Fractionation Assay 

To determine the changes in localization of AhR and β-catenin, treated shCtrl and shIDO1 HuH-7 cells were fractionated into nuclear and cytoplasmic fractions using NE-PER^TM^ Nuclear and Cytoplasmic Extraction Reagent (Thermo Fisher Scientific, Rockford, IL, USA) according to the manufacturer’s instructions. Subsequently, fractionated samples were analyzed via immunoblotting to establish AhR and β-catenin patterns. The cytoplasmic marker β-actin and nuclear marker lamin B were used as fractionation purity controls.

### 4.6. Co-Immunoprecipitation (Co-IP)

Cell lysates of shCtrl and shAhR HuH-7 after treatment with IFN-γ (40 ng/mL) or KYN (100 μM) for 8 h were harvested as described above and pre-cleaned with protein G beads (GE Healthcare Life Science, Chicago, IL, USA) at 4 °C for 4 h. Protein G beads coupled with antibodies for IgG or AhR (Santa Cruz, USA) were added to pre-cleaned lysates and incubated at 4 °C for 4 h to immunoprecipitate AhR and interacting molecules. Bound proteins were eluted and analyzed via SDS-PAGE and immunoblotting as described above.

### 4.7. Chromatin Immunoprecipitation (ChIP) Assay

shCtrl and shIDO1 HuH-7 cells were treated with IFN-γ (40 ng/mL) or KYN (100 μM) for 8 h. Cells were harvested, washed with PBS, and crosslinked with 1% formaldehyde [60]. Next, cells were lysed and chromosomes fragmented in 1 mL lysis buffer containing 50 mM Tris–HCl, 10 mM EDTA, and 1% SDS, pH 8.0. Control IgG and antibodies against AhR (Santa Cruz) or β-catenin (BD Biosciences) were used for immunoprecipitation of DNA-protein complexes, which were further pulled down with Protein A/G resin beads (GE Healthcare Life Science). Immunoprecipitated DNA was retrieved and purified. Gene-specific primers for Snail promotor were used to determine the binding status of AhR [32]. Input (20%) control was loaded. Primer sequences are presented in Table S1.

### 4.8. Statistical Analysis

Cell proliferation, viability, migration, and invasion assays were performed at least three times. The representative results are shown. The two-tailed independent samples *t* test with assumption of independence was used for analysis of significant differences among groups unless mentioned elsewhere, with the level of statistical significance set at * *p* < 0.05, ** *p* < 0.01 or *** *p* < 0.001. Values obtained for all measurements are expressed as means ± SD.

## Data Availability

The data presented in this study are available on request from the corresponding author.

## Supplementary Materials

The following are available online at https://www.mdpi.com/article/10.3390/ijms222111661/s1.

## Abbreviations

HCC	Hepatocellular carcinoma
EMT	Epithelial–mesenchymal transition
IDO1	Indoleamine 2,3-dioxygenase 1
IFN-γ	Interferon-γ
AhR	Aryl hydrocarbon receptor
KYN	Kynurenine
PI3K	Phosphoinositide 3-kinases
Akt	Protein kinase B
CYP1A1	Cytochrome P450 1A1
PTEN	Phosphatidylinositol 3,4,5-trisphosphate 3-phosphatase and dual-specificity protein phosphatase PTEN
Src	Proto-oncogene tyrosine-protein kinase Src
GSK-3β	Glycogen synthase kinase-3 beta
c-myc	Myc proto-oncogene protein
cyclin D1	G1/S-specific cyclin-D1
p53	Cellular tumor antigen p53
p21^WAF/Cip1^	Cyclin-dependent kinase inhibitor 1
p27^Kip^	Cyclin-dependent kinase inhibitor 1B
E-cadherin	Cadherin-1
Snail	Zinc finger protein SNAI1
ZEB-1	Zinc finger E-box-binding homeobox 1
