# Supplementary material for: Aberrant Upregulation of Indoleamine 2,3-Dioxygenase 1 Promotes Proliferation and Metastasis of Hepatocellular Carcinoma Cells via Coordinated Activation of AhR and β-Catenin Signaling
Source: Int J Mol Sci. 2021 Oct 28;22(21):11661. doi: 10.3390/ijms222111661 (PMC8583706; doi:10.3390/ijms222111661)
Supplement: Supplementary file 1 [file ijms-22-11661-s001.zip › ijms-1240731-supplementary.pdf]

Supplemental information

Aberrant Upregulation of Indoleamine 2,3-Dioxygenase 1 Promotes Proliferation and Metastasis of Hepatocellular Carcinoma Cells via Coordinated Activation of AhR and β-Catenin Signaling

Chih-Ta Chen[1], Pei-Hua Wu[1], Chia-Chi Hu[1], Hsiao-Ching Nien[2,5], Jin-Town Wang[3,4], Jin-Chuan Sheu[4,5], Lu-Ping Chow[1]

From the [1] Graduate Institute of Biochemistry and Molecular Biology, College of Medicine, National Taiwan University, Taipei, Taiwan; [2] Department of Family Medicine, National Taiwan University Hospital, Taipei, Taiwan. [3] Department of Microbiology, College of Medicine, National Taiwan University, Taipei, Taiwan. [4] Department of Internal Medicine, National Taiwan University Hospital, College of Medicine, National Taiwan University, Taipei, Taiwan; [5] Liver Disease Prevention and Treatment Research Foundation, Taipei, Taiwan

* Correspondence: Lu-Ping Chow; chowip@ntu.edu.tw

Supplemental Table S1. shRNA target and gene-specific primer sequences

Gene	shRNA target sequence / primer sequence
IDO1	CGCTGTTGGAAATAGCTTCTT
AhR	ATCCACAGTCAGCCATAATAA
Snail promoter	F: 5'- CGGCGGAGACGAGCCTCCGATTG-3'<br>R: 5'- GGAAAGAGCGCGGCATAGTGG-3'

F, forward; R, reverse

Supplemental Table S2. List of antibodies used in this study

**Antibody**	**Manufacturer**
Src (CST#2108), p-Src (CST#2101), p-GSK-3β (CST#9323),	Cell Signaling Technology, USA
AhR (sc-133088), Akt (sc-8312), p-Akt (sc-7985), c-myc (sc-40), p53 (sc-56182), β-actin (sc-47778)	Santa Cruz Biotechnology, USA
PTEN (GTX101025), GSK-3β (GTX111192), CyclinD1 (GTX108624), $p27^{Kip1}$ (GTX27961), Snail (GTX100754), fibronectin (GTX112794), ZEB-1 (GTX105278)	GeneTex, USA
E-cadherin (MA5-15711), $p21^{WAF1/Cip1}$ (MS-891)	Thermo Fisher Scientific, USA
IDO1 (ab55305)	Abcam, UK
β-catenin (BD610153)	BD Bioscience, USA

Supplemental Figure Legends

Supplemental Figure S1. Establishment of the HuH-7 IDO1 activity induction model. (A) HuH-7 cells were infected with lentiviruses containing shIDO1 or control shRNA and treated with indicated doses of IFN-γ for 48 h, lysed and analyzed for IDO1 expression via immunoblotting. **(B)** IDO1 activity was evaluated via analyzing kynurenine production in the condition medium. Media were harvested after control and IDO1 knockdown HuH-7 cells were treated with or without IFN-γ (40 ng/ml) for 48 h and kynurenine levels measured via colorimetric assay. Quantitative data are presented as mean ± SD (shCtrl, control shRNA; shIDO1, shRNA against IDO1; **, $p < 0.01$).

Supplemental Figure S2. Upregulation of IDO1 activity promotes proliferation, migration and invasion of HCC cells. (A) HCC cell lines were treated with IFN-γ (40 ng/ml) for 48 h, lysed and analyzed for IDO1 expression via immunoblotting. **(B)** Knockdown of IDO1 significantly suppressed the proliferation of Sk-Hep1, while KYN slightly recued the growth of shIDO1 cells. Viability of IDO1 knockdown and control Sk-Hep1 cells under IFN-γ (40 ng/ml) treatment with or without KYN (100 μM) rescue monitored for indicated days via MTT assay. Plots depict relative proliferation fold versus number of days. **(C)**. The growth of shCtrl, shIDO1 with or without KYN rescue showed no significant difference in the 3-day interval. Viability of IDO1 knockdown

and control Ph5Ch8 cells under IFN-γ (40 ng/ml) treatment with or without KYN (100 μM) rescue monitored for indicated days via MTT assay. Plots depict relative proliferation fold versus number of days. **(D)** Knockdown of IDO1 significantly inhibited the migration and invasion of Sk-Hep1 compared to the shCtrl cells. KYN rescued the migration of shIDO1 cells while the rescue of invasion was not prominent. Upper, Transwell migration assay of shCtrl and shIDO1 Sk-Hep1 cells treated with IFN-γ (40 ng/ml) with or without KYN (100 μM) rescue. Cells in the central field of each insert visualized via light microscopy after 48 h. Lower, Transwell invasion assay of control and IDO1 knockdown Sk-Hep1 cells under IFN-γ (40 ng/ml) treatment with or without KYN (100 μM) rescue. Cells in the central field of each insert visualized via light microscopy after 48 h. **(E)** Knockdown of IDO1 didn't impact the migration and invasion of Ph5Ch8 among the shCtrl, shIDO1 and rescued group. Upper, Transwell migration assay of shCtrl and shIDO1 Ph5Ch8 cells treated with IFN-γ (40 ng/ml) with or without KYN (100 μM) rescue. Cells in the central field of each insert visualized via light microscopy after 48 h. Lower, Transwell invasion assay of control and IDO1 knockdown Ph5Ch8 cells under IFN-γ (40 ng/ml) treatment with or without KYN (100 μM) rescue. Cells in the central field of each insert visualized via light microscopy after 48 h (shCtrl, control shRNA; shIDO1, shRNA against IDO1; n.s., not significant; *, $p < 0.05$; **, $p < 0.01$; ***, $p < 0.001$).

Supplemental Figure S3. AhR and β-catenin nuclear translocation, activity and mutual interactions are IDO1- and AhR activity-dependent. (A) IFN-γ significantly induced nuclear translocation of AhR in the shCtrl HuH-7 cells. Knockdown of IDO1 impaired nuclear translocation of AhR in the shIDO1 HuH-7 cells while KYN rescue restored the nuclear translocation of AhR. Control and IDO1 knockdown HuH-7 cells were treated with or without IFN-γ (40 ng/ml) for 48 h with or without KYN (100 μM) rescue and AhR localization examined via IF staining. Cell nuclei were stained with DAPI (blue) and AhR with FITC (green). **(B)** KYN dose-dependently induced nuclear translocation of AhR in the shCtrl HuH-7 cells. shCtrl HuH-7 cells were treated with KYN at the indicated concentrations and AhR localization examined via IF staining. Cell nuclei were stained with DAPI (blue) and AhR with FITC (green). **(C)** KYN simultaneously induced nuclear translocation of AhR and β-catenin in a dose-dependent manner in the shCtrl HuH-7 cells. Control HuH-7 cells were treated the indicated concentrations of KYN, and AhR and β-catenin localization examined via IF staining. Cell nuclei were stained with DAPI (blue), AhR with FITC (green) and β-catenin with TRITC (red). **(D)** Examination of nuclear translocation of AhR and β-catenin in shCtrl HuH-7 cells under KYN (100 μM) stimulation for the indicated times using the cell fractionation assay. KYN promoted nuclear AhR increase in a time-dependent manner. Upper, fractionated samples were separated via SDS-PAGE and analyzed via

immunoblotting with the specified antibodies. Lower, relative changes in nuclear localization of AhR and β-catenin were quantified using Lamin B as normalization control. **(E)** Evaluation of AhR and β-catenin activity in control HuH-7 cells under KYN (100 μM) induction for the indicated times based on downstream CYP1A1 and p-β-catenin (S33/37/T41) expression levels via immunoblotting, upper. CYP1A1 increased time-dependently under KYN stimulation while p-β-catenin (S33/37/T41) decreased. Lower, relative changes in CYP1A1 and p-β-catenin were quantified using β-actin or β-catenin as the normalization control respectively. **(F)** Interactions between AhR and β-catenin induced by the IDO1 activity-derived product, KYN (100 μM), were validated using a co-immunoprecipitation assay and subsequent immunoblot analysis. Compared to shCtrl HuH-7 cells, co-immunoprecipitated β-catenin with AhR was significantly decreased in the shAhR cells. All statistical data were calculated from three independent replicates (shCtrl, control shRNA; shIDO1, shRNA against IDO1; shAhR, shRNA against AhR; *, $p < 0.05$; **, $p < 0.01$; ***, $p < 0.001$).

Supplemental Figure S4. IDO1 activation induces downstream AhR and β-catenin nuclear translocation and activity in Sk-Hep1 cells. (A) IFN-γ stimulation increased the nuclear localization of AhR in the shCtrl Sk-Hep1 cells. Knockdown of IDO1 inhibited nuclear translocation of AhR in the shIDO1 Sk-Hep1 cells while KYN rescue partially restored the nuclear translocation of AhR. Control and IDO1 knockdown Sk-

Hep1 cells were treated with or without IFN-γ (40 ng/ml) for 48 h in the presence or absence of KYN (100 μM), and AhR and β-catenin localization examined via IF staining. Cell nuclei were stained with DAPI (blue), AhR with TRITC-labeled antibody (red) and β-catenin with FITC-labeled antibody (green). **(B)** Examination of AhR and β-catenin activity under IFN-γ (40 ng/ml) induction in control, shIDO1 or KYN (100 μM) rescued Sk-Hep1 cells by comparing downstream CYP1A1 and p-β-catenin (S33/37/T41) expression via immunoblotting. Induction of IDO1 increased the expression of CYP1A1 and inhibited the phosphorylation of β-catenin (S33/37/T41) in the control cells compared to the shIDO1 counterparts. Rescue by KYN partially restored the effect in the shIDO1 cells.

Supplemental Figure S5. β-Catenin activation by GSK-3β inhibition via Src-PTEN-Akt activation is AhR-dependent. (A) KYN inhibited the expression of PTEN and suppressed the phosphorylation of Akt(S473) and GSK-3β(S9) in a time-dependent manner. Left, shCtrl HuH-7 cells were treated with KYN (100 μM) for the indicated times. Cell lysates were separated via SDS-PAGE and analyzed using immunoblot analysis. Right, quantitative analysis of alterations in expression levels of PTEN, p-Akt and p-GSK-3β. **(B)** Activation of β-catenin by the PTEN-Akt-GSK-3β axis is AhR activity-dependent. PTEN was upregulated in the shAhR cells under KYN induction relative to the controls, left. The phosphorylation of Akt(S473) and GSK-3β(S9) was

inhibited under KYN induction in the AhR knockdown cells compared to the controls, right. Expression levels of PTEN, p-Akt and p-GSK-3β in control and AhR knockdown HuH-7 cells under KYN (100 μM) stimulation were examined by immunoblotting. **(C)** Activation of Src by AhR after stimulation of IDO1 activity. KYN time-dependently increased the phosphorylation of Src (Y416) in HuH-7 cells. Upper, control HuH-7 cells were stimulated with KYN (100 μM) for the indicated times. Cell lysates were separated via SDS-PAGE and variations in p-Src status analyzed using immunoblotting. Lower, quantitative changes in phosphorylation levels of Src. Intensity was quantified via densitometry and normalized to that of β-actin (shCtrl, control shRNA; shAhR, shRNA against AhR; *, $p < 0.05$; **, $p < 0.01$; ***, $p < 0.001$).

Supplemental Figure S6. Modulation of AhR-mediated Src-PTEN-Akt-GSK-3β pathway under IDO1 activation in HCC and normal hepatocyte cells. Sequential induction of IDO1 and AhR activity inhibited GSK-3β via Src activation, PTEN suppression and Akt activation in HCC but not normal cells. **(A)** The expression of PTEN was downregulated while the phosphorylation of Src(Y416), Akt(S473) and GSK-3β(S9) was upregulated under IDO1 induction by IFN-γ in the shCtrl cells or AhR activation by KYN rescue in shIDO1cells. shCtrl and shIDO1 Sk-Hep1 cells were induced by IFN-γ (40 ng/ml) with or without KYN (100 μM) rescue. Cell lysates were separated via SDS-PAGE and analyzed via immunoblotting with the specified

antibodies. **(B)** The levels of PTEN, p-Src(Y416), p-Akt(S473) and p-GSK3β(S9) remained unaltered in Ph5Ch8 cells. shCtrl and shIDO1 Ph5Ch8 cells were induced by IFN-γ (40 ng/ml) with or without KYN (100 μM) rescue. Cell lysates were separated via SDS-PAGE and analyzed via immunoblotting with the specified antibodies (shCtrl, control shRNA; shIDO1, shRNA against IDO1).

Supplemental Figure S7. Modulation of proliferative and EMT-related genes is IDO1 and AhR-dependent. (A) Expression levels of c-myc and cyclin D1 under AhR activation after IDO1 induction. C-myc, cyclinD1 were upregulated time-dependently by KYN induction in HuH-7 cells. Upper, control HuH-7 cells were treated with KYN (100 μM) for the indicated times and expression of the indicated proteins analyzed via immunoblotting. Lower, quantitative analysis of variations in c-myc and cyclin D1 levels. **(B)** Evaluation of expression of the EMT regulator, Snail, and epithelial marker, E-cadherin, under AhR activation. Treatment of KYN increased Snail and decreased E-cadherin expression in a time-dependent manner in HuH-7 cells. Upper, control HuH-7 cells were treated with KYN (100 μM) for the indicated times. Samples were separated using SDS-PAGE and analyzed via immunoblotting. Lower, quantitative analysis of variations in Snail and E-cadherin levels. **(C)** The expression of ZEB-1 and fibronectin was increased time-dependently by KYN induction. Immunoblot analysis of two Snail-regulated mesenchymal markers, ZEB-1 and fibronectin, under AhR

activation, upper. Lower, quantification of alterations in ZEB-1 and fibronectin expression. All statistical data were calculated from three independent replicates. **(D)** Expression levels of proliferative markers, c-myc and cyclin D1, the EMT regulator, Snail, and epithelial marker, E-cadherin under IDO1 and AhR activation. CyclinD1 and Snail were upregulated under IDO1 induction in shCtrl cells while E-cadherin was downregulated compared to the shIDO1 cells. Control and shIDO1 Sk-Hep1 cells were treated with IFN-γ (40 ng/ml) with or without KYN (100 μM) rescue, and expression of the indicated proteins was analyzed via immunoblotting with specific antibodies. **(E)** Immunoblot analysis of expression levels of two Snail-regulated mesenchymal markers, ZEB-1 and fibronectin in Sk-Hep1 cells, under IDO1 and AhR induction. Only fibronectin was significantly increased under IDO1 induction in the shCtrl cells. Quantitative data were calculated from three independent replicates (*, $p < 0.05$; **, $p < 0.01$; ***, $p < 0.001$).

Supplemental Figure S8. IDO1 and AhR activation modulate checkpoint proteins to facilitate cell proliferation. (A) Expression levels of checkpoint proteins p53, $p21^{WAF1/Cip1}$ and $p27^{Kip1}$ under IDO1 activation were evaluated. All three checkpoint inhibitors were inhibited in shCtrl HuH-7 cells relative to shIDO1 counterparts. KYN restored the inhibition in the shIDO1 cells. Left, shCtrl and shIDO1 HuH-7 cells were treated with IFN-γ (40 ng/ml) with or without KYN (100 μM) rescue and expression of

the indicated proteins analyzed via immunoblotting. Right, quantitative analysis of variations in the indicated proteins. **(B)** Evaluation of expression of the checkpoint proteins under AhR activation. KYN time-dependently suppressed the expression of p53, p21 and p27 in control HuH-7 cells. Upper, control HuH-7 cells were treated with KYN (100 μM) for the indicated times. Samples were separated using SDS-PAGE and analyzed via immunoblotting. Lower, quantitative analysis of variations in checkpoint protein expression. All statistical data were calculated from three independent replicates. **(C)** Expression levels of checkpoint proteins p53, $p21^{WAF1/Cip1}$ and $p27^{Kip1}$ in Sk-Hep1 cells under IDO1 activation were evaluated. Induction of IDO1 significantly decreased $p27^{Kip1}$ in the shCtrl cells compared to the shIDO1 counterparts. shCtrl and shIDO1 Sk-Hep1 cells were treated with IFN-γ (40 ng/ml) with or without KYN (100 μM) rescue and expression of the indicated proteins analyzed via immunoblotting (shCtrl, control shRNA; shIDO1, shRNA against IDO1; *, $p < 0.05$; **, $p < 0.01$; ***, $p < 0.001$).

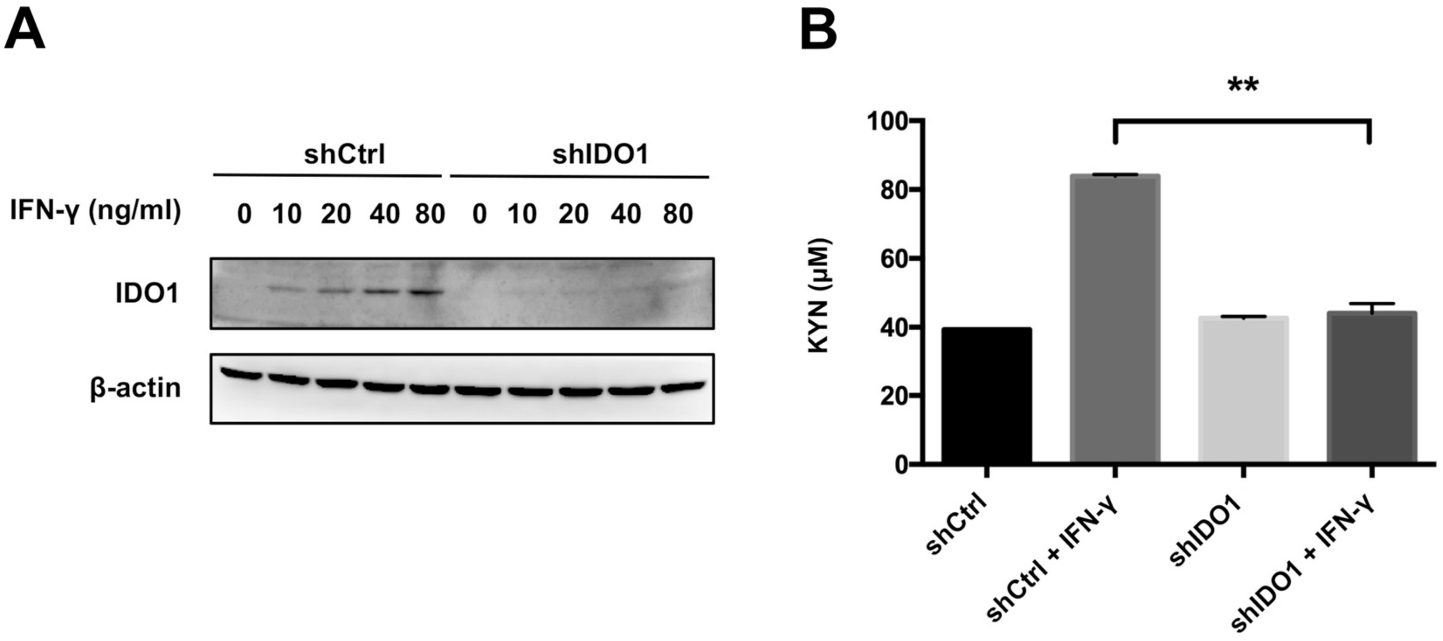

Supplemantal Figure S1

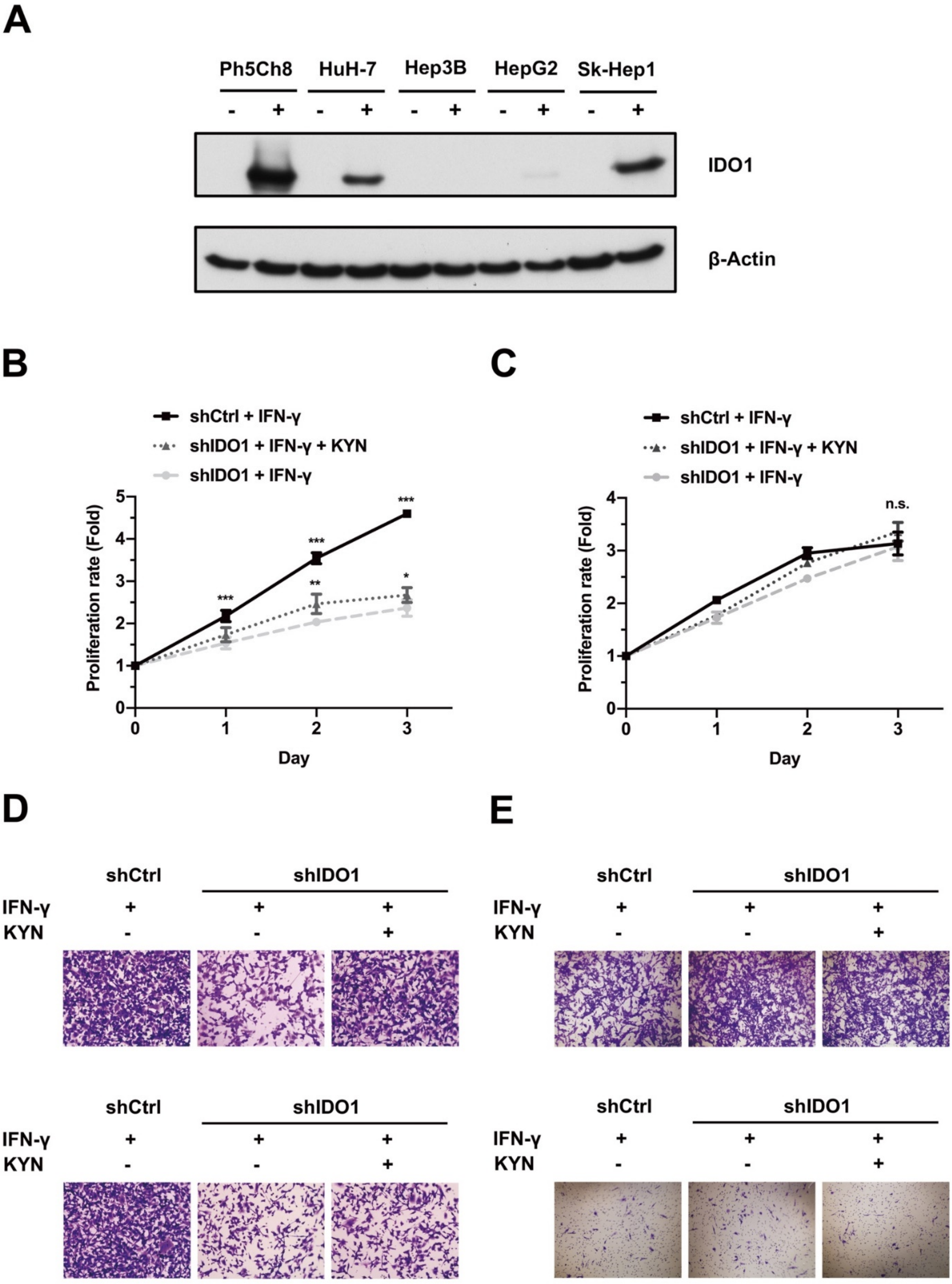

Supplemantal Figure S2

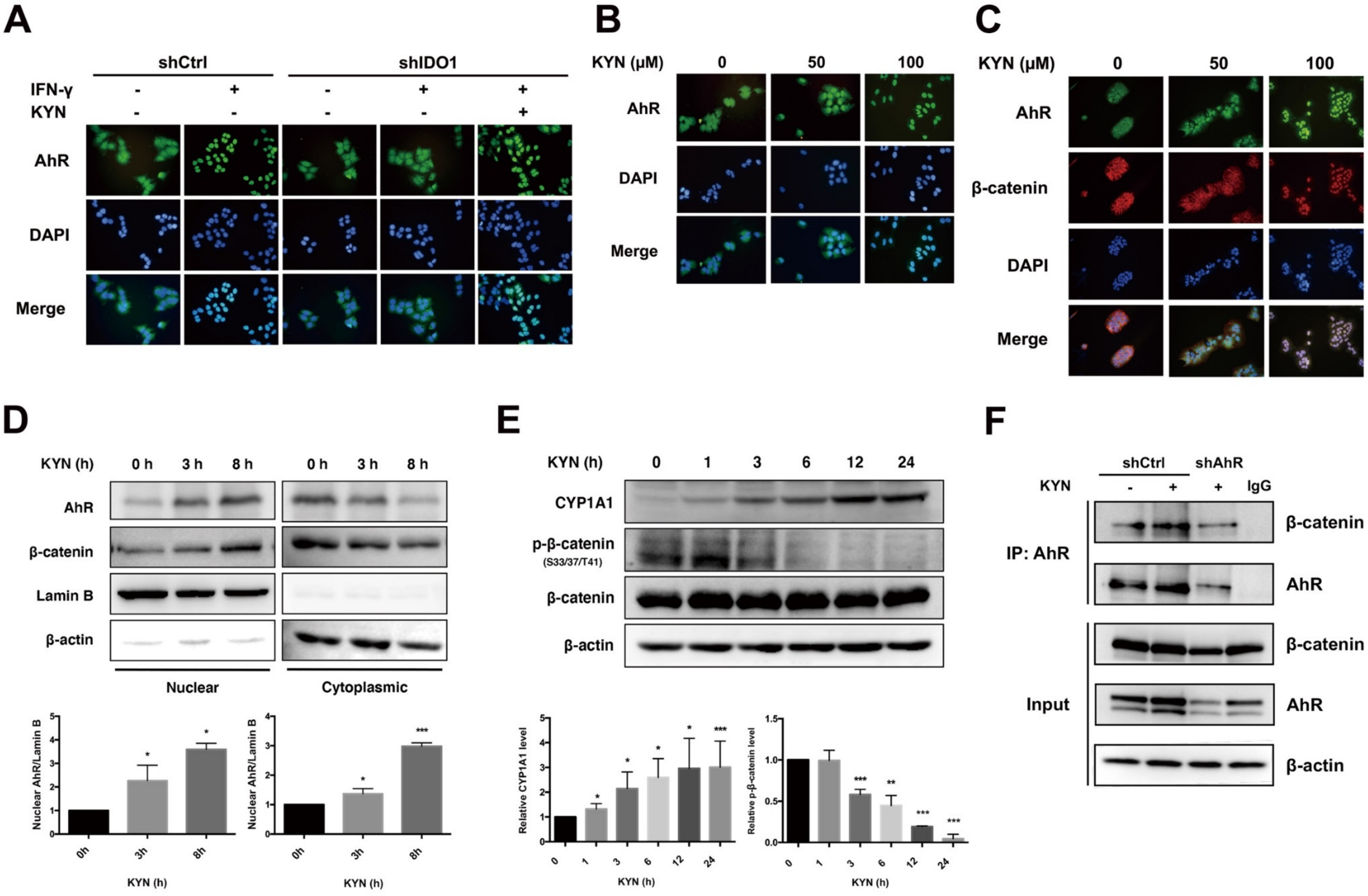

Supplemantal Figure S3

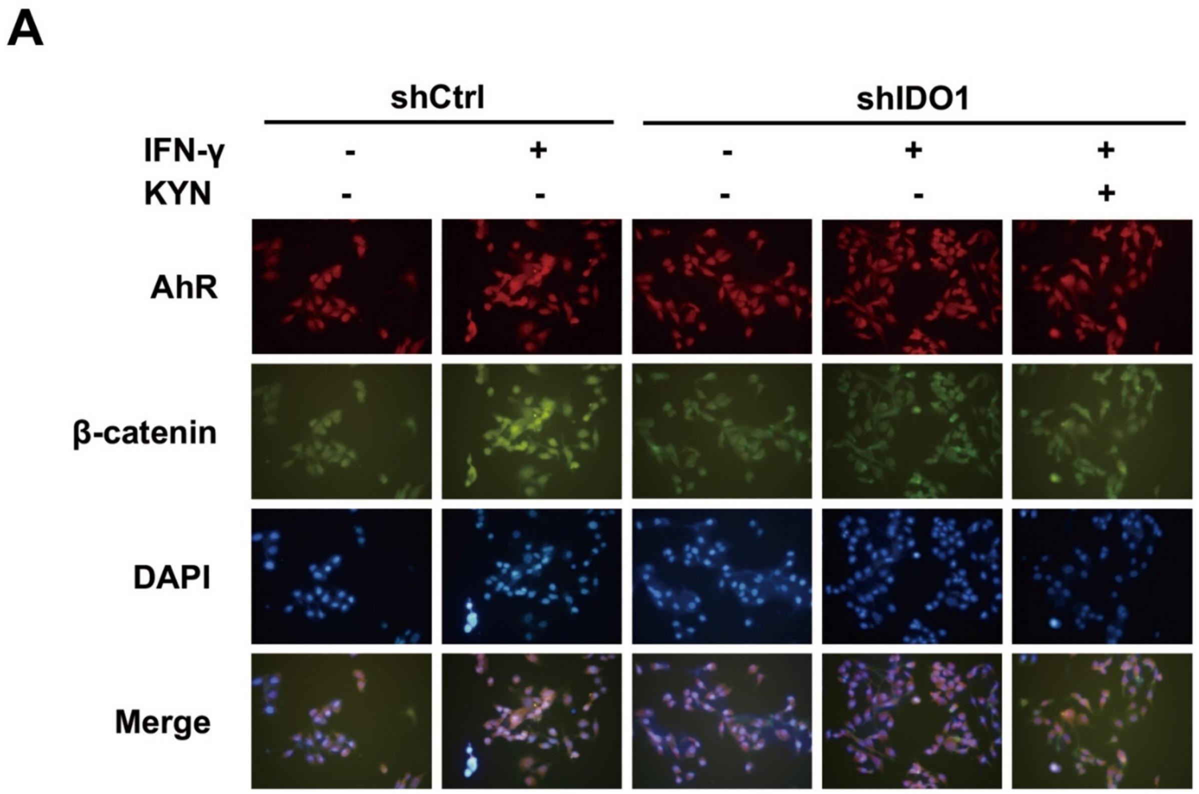

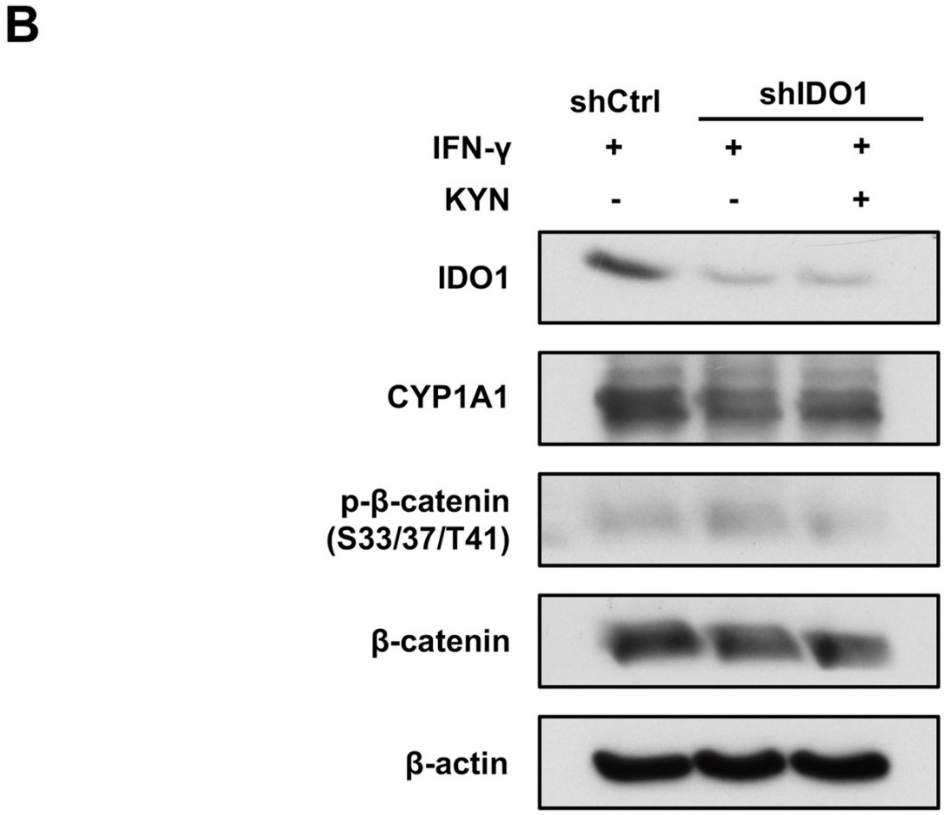

Supplemantal Figure S4

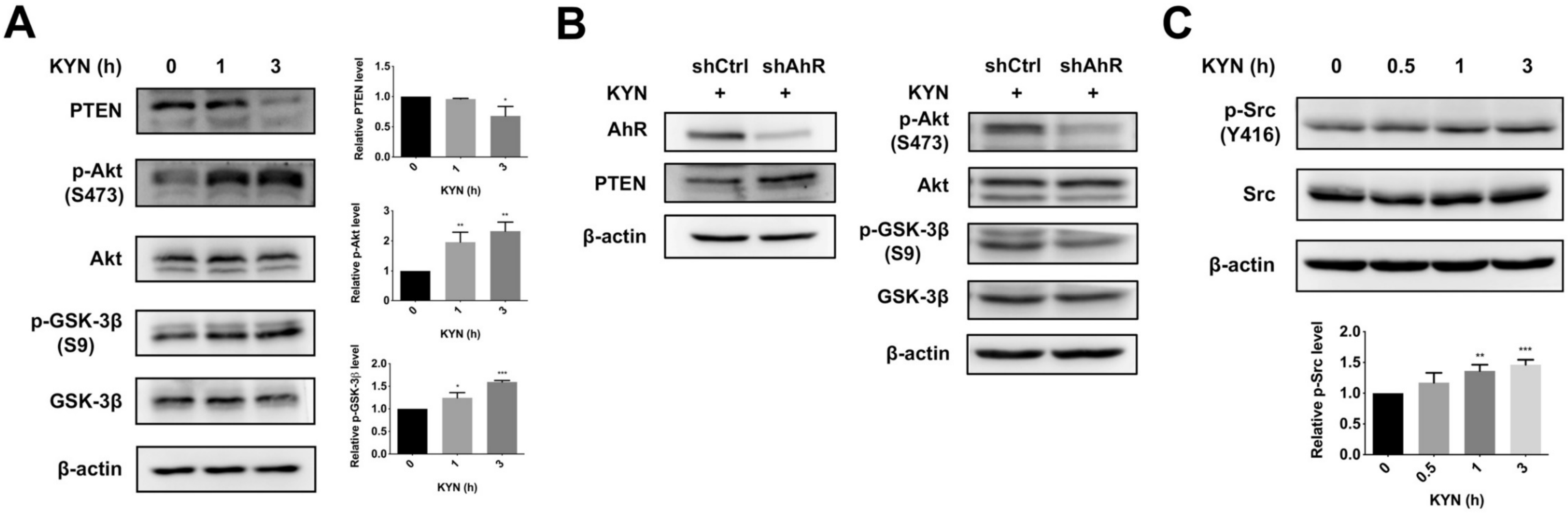

Supplemantal Figure S5

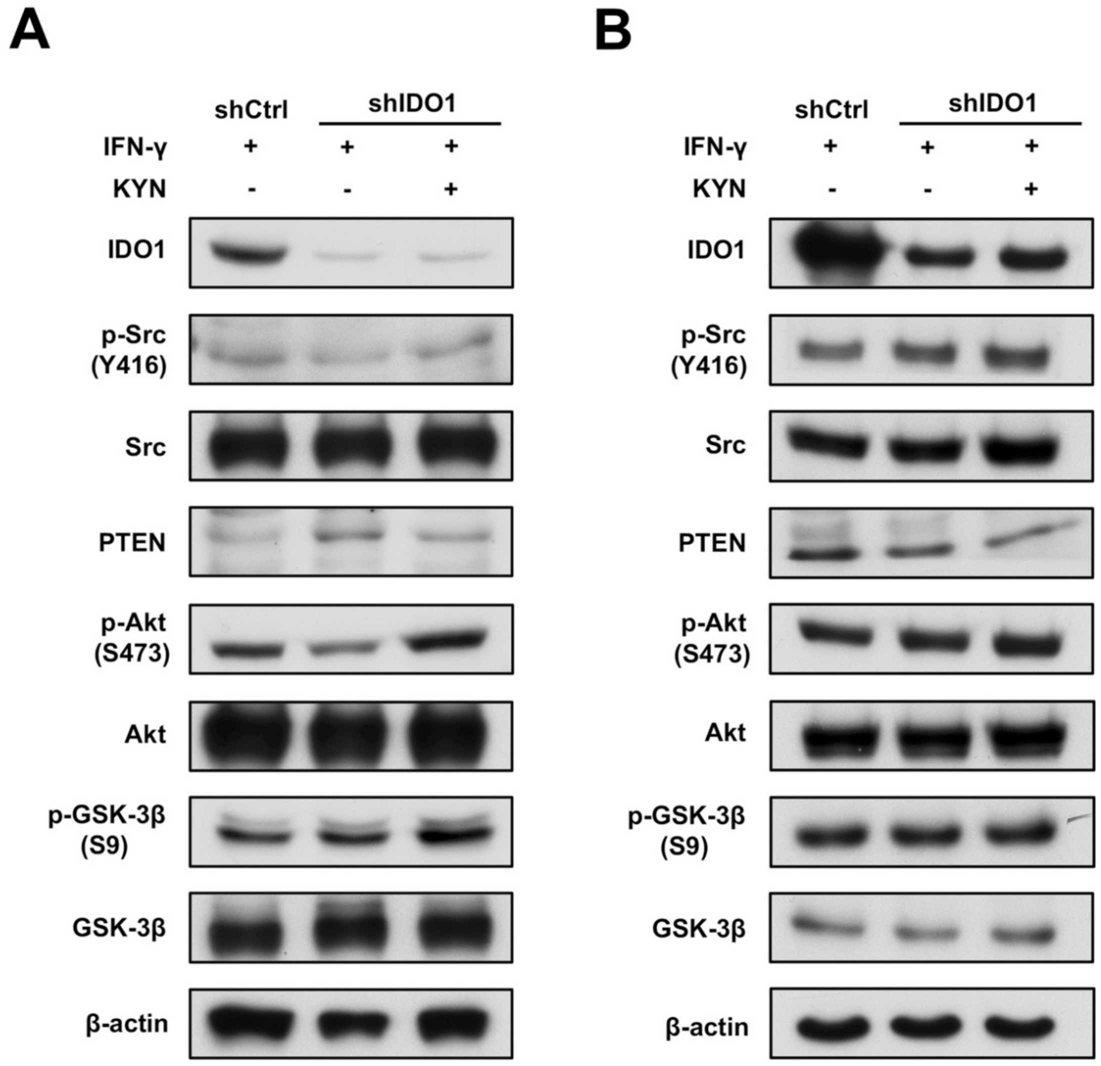

Supplemantal Figure S6

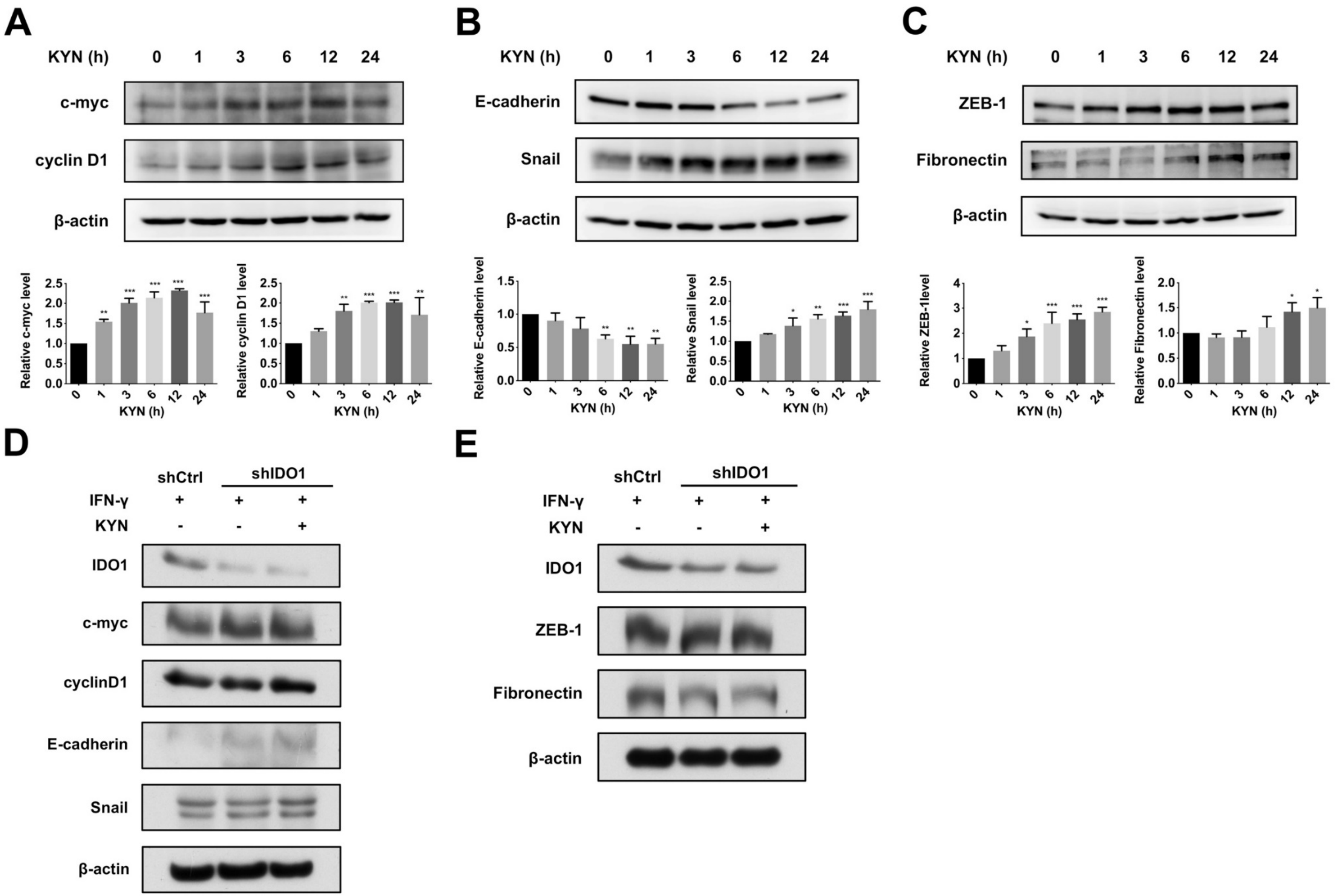

Supplemantal Figure S7

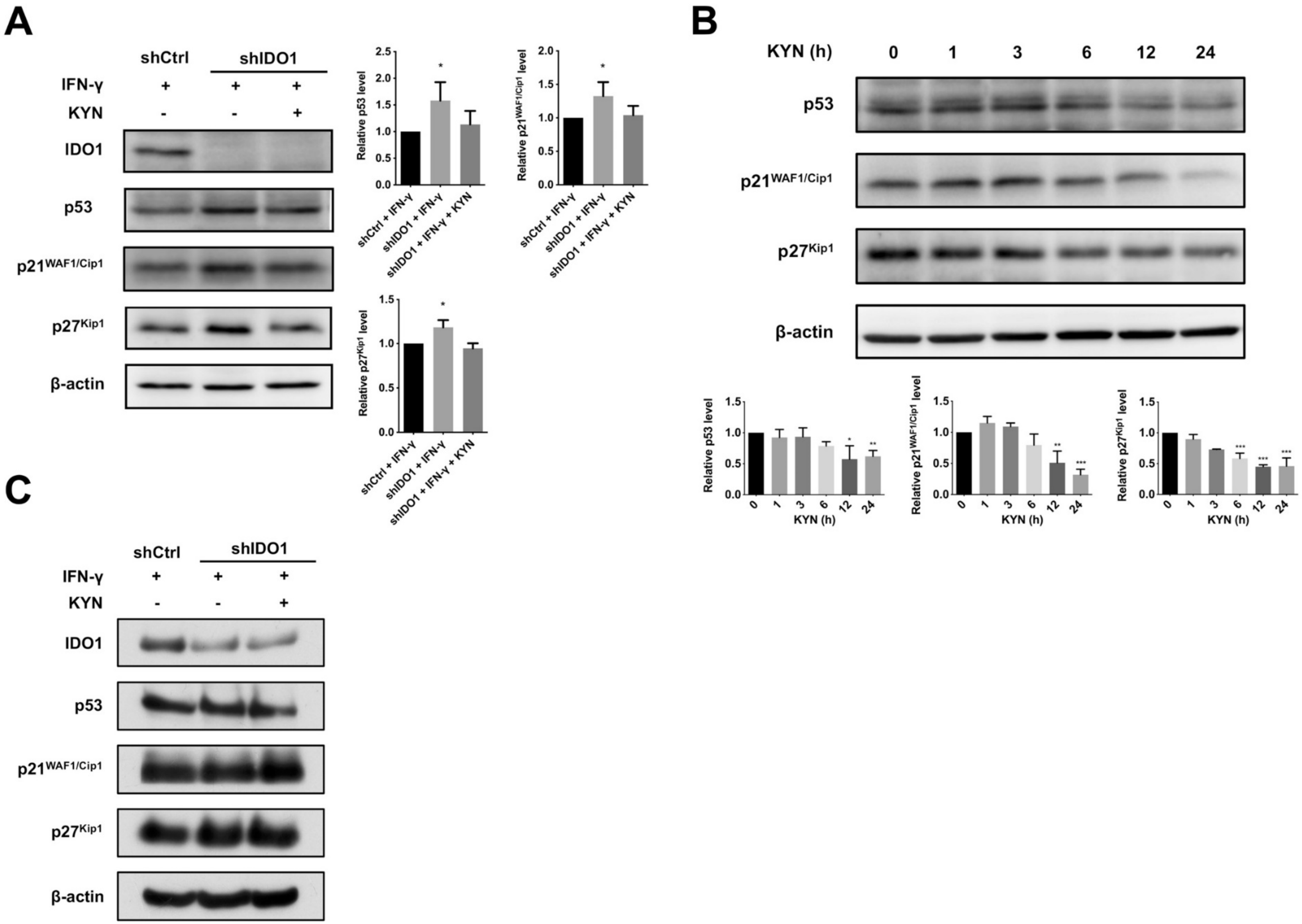

Supplemantal Figure S8
